# Hydrothermal Synthesis of Ultra-Light Coal-Based Graphene Oxide Aerogel for Efficient Removal of Dyes from Aqueous Solutions

**DOI:** 10.3390/nano8090670

**Published:** 2018-08-29

**Authors:** You Lv, Baolin Xing, Mingkun Zheng, Guiyun Yi, Guangxu Huang, Chuanxiang Zhang, Ruifu Yuan, Zhengfei Chen, Yijun Cao

**Affiliations:** 1 Henan Key Laboratory of Coal Green Conversion, College of Chemistry and Chemical Engineering, Henan Polytechnic University, Jiaozuo 454003, China; lvyou0819@163.com (Y.L.); ygyun@hpu.edu.cn (G.Y.); hgxu@hpu.edu.cn (G.H.); zcx223@hpu.edu.cn (C.Z.); yrf@hpu.edu.cn (R.Y.); 2 Henan Province Industrial Technology Research Institute of Resources and Materials, Zhengzhou University, Zhengzhou 450001, China; 3 School of Science, Hubei University of Technology, Wuhan 430068, China; zhengmingkun1993@163.com; 4 Graduate School of Energy Science, Kyoto University, Yoshida, Sakyo-ku, Kyoto 606-8501, Japan; zhengfei.chen@gmail.com

**Keywords:** coal-based graphene oxide, aerogel, adsorption, dyes, carboxymethyl cellulose

## Abstract

A novel carboxymethyl cellulose (CMC)-supported graphene oxide aerogel (CGOA) was fabricated from a cost-effective and abundant bituminous coal by a mild hydrothermal process and freeze-drying treatment. Such an aerogel has cross-linked graphene oxide layers supported by CMC, and therefore, displays high mechanical strength while having ultra-low density (8.257 mg·cm^−3^). The CGOA has a 3D interconnected porous structure, beneficial graphene framework defects and abundant oxygen-containing functional groups, which offer favorable diffusion channels and effective adsorption sites for the transport and adsorption of dye molecules. The adsorption performance of rhodamine B by an optimized CGOA shows a maximum monolayer adsorption capacity of 312.50 mg·g^−1^, as determined by Langmuir isotherm parameters. This CGOA exhibited a better adsorption efficiency (99.99%) in alkaline solution, and satisfactory stability (90.60%) after three cycles. In addition, adsorption experiments on various dyes have revealed that CGOA have better adsorption capacities for cationic dyes than anionic dyes.

## 1. Introduction

Water pollution has become one of the most urgent global environmental issues due to the rapid development of modern industry worldwide. Among a variety of water pollutions, dyes are the most common pollutants appearing in wastewater from the textile, printing, paper, cosmetics, feather, and leather industries [1,2,3,4]. Since most dyes do not biodegrade and have high toxicity (e.g., carcinogenic and mutagenic effects), the presence of dyes even at very low concentrations (less than 1 ppm for some dyes) in water is highly visible and undesirable, and can lead to severe problems in human health [4,5,6]. Many techniques and processes have been applied for the treatment of dyes from wastewater, including photocatalytic degradation [7], membrane separation [8], chemical precipitation [9], biological oxidation [10], and adsorption [11]. Among these methods, adsorption is considered an effective and promising approach, because it has the advantages of low-cost, convenient operation, no secondary pollution, and recyclability [12,13]. Therefore, the exploitation of cost-effective adsorbents with high efficiency is an important focus in related research fields. 

Graphene, as a new type of carbon nanomaterial with large specific surface area and excellent physical and chemical properties [14], has been widely used in adsorption for the waste water treatment [15,16,17] and biomedical fields [18,19,20,21,22]. Graphene oxide (GO) has similar properties to graphene, but is more hydrophilic because of a variety oxygen functional groups, which is a precondition as an adsorbent used in aqueous systems [23,24]. However, in the past few years, the potential toxicity of graphene/GO has attracted much attention from scholars in various fields [25,26]. The particulate state, surface functional groups, and oxygen content/surface charges of graphene/GO may significantly affect its toxicity in biological systems [27]. The application of GO aerogels has largely broken through the above limitations owing to its unique macrostructure with a block backbone and very low density for facile separation from aqueous solutions. In addition, GO aerogel has a highly active surface and large pore volume with three-dimensional (3D) interconnected porous structures, which can offer multi-dimensional adsorption active sites to ensure large adsorption capacity and desirable adsorption efficiency. Abundant hydrophilic polar groups such as hydroxyl, carboxyl, and epoxy groups distributed on the surface of carbon frameworks improve the affinity of GO aerogels to adsorbates in aqueous solution [28,29,30,31,32].

Several synthetic techniques, including self-assembly [33], bioassembly [34], unidirectional freezing [35], and ice crystal templating [32], have been developed to prepare GO aerogel for wastewater treatment. However, these GO aerogels with 3D architectures often suffer from poor mechanical strength, and are prone to collapses or even fragmentation upon immersion in water, which not only hinders the effective reutilization of the aerogel, but causes secondary pollution in water environments. In order to overcome this drawback, introducing various types of cellulose as reinforcement agents or cross-linking agents during the preparation process has been studied recently as a means to improve the mechanical strength and stability of the GO aerogel. Sun et al. used bamboo as the starting material to synthesize GO/cellulose aerogels for antibiotics removal by a one-step ultrasonication method under two conditions [36,37]. Tehrani et al. prepared composite hydrogel for cationic dye removal via sonicating GO and cellulose nanowhiskers in dimethylformamide, and continuously stirring at 110 °C for 2 days [38]. Wan et al. reported GO/cellulose hybrid aerogels prepared through a solution mixing-regeneration-freeze drying process [39]. Limited raw materials, toxic reagents, long production cycles and complicated preparation processes limit large-scale practical application, despite these composite materials exhibiting satisfactory adsorption capacities and structural stability. Therefore, it is highly desirable to develop a facile and cost-effective strategy to fabricate GO aerogel with a high adsorption capacity and excellent mechanical properties.

Carboxymethyl cellulose (CMC) is a water-soluble anionic linear polysaccharide derived from natural cellulose which is considered an inexpensive biocompatible material [40], and which usually contains large amounts of active functional groups including hydroxyl and carboxyl groups that act as desirable active sites during adsorption process [41]. In addition, the high specific surface area and abundant oxygen-containing functional groups of GO mean that they are easily functionalized by CMC due to the strong H-bond interaction between individual GO nanosheets and CMC [42]. Therefore, CMC/GO composite materials have attracted attention as effective adsorbents for the removal of heavy metal ions and organic dyes from aqueous solution. Zhang Y. et al. prepared CMC/GO monoliths (GO, 0.2~5.0 wt %) by a unidi-rectional freeze-drying method, and proved that the adsorption efficiency of multiple heavy metal ions was improved [43]. Liu J. et al. fabricated CMC/GO hydrogel microparticles (GO, 2~8 wt %) via spray drying, and found that these hydrogel microparticles can effectively improve the adsorption capacity of dye molecules (59 mg·g^−1^ for methylene blue and 66 mg·g^−1^ for Eosin Y) [44]. Varaprasad K. et al. conducted preparation on dye removal CMC-Acrylamide-GO hydrogels (GO, 0.05~0.14 wt %) by a free-radical polymerization method; the resulting maximum acid blue absorption capacity was 185.45 mg·g^−1^ [45]. Although these methods have successfully synthesized various 3D CMC/GO composite materials, and the introduction of GO contributes significantly to the adsorption properties of the material, surprisingly, little attention has been devoted to the effects of a high quality proportion of graphene on the adsorption properties of materials. 

In this work, GO is first obtained by a modified Hummers method using synthetic graphite derived from earth-abundant and low-cost bituminous coal as starting precursor. By comparison with conventional GO from natural graphite, coal-based graphene oxide (CGO) possesses higher porosity and larger surface area, because such synthetic graphite has relatively more structural defects and stronger binding forces among the neighboring graphite layers, which may provide more adsorption sites in the carbon matrix. Different contents of CMC are then incorporated into the CGO solution, and aerogels are prepared through a hydrothermal reaction coupled with freeze-drying. The microstructure and surface chemical state of the obtained final products were investigated through various characterization techniques. The adsorption behaviors of such a graphene oxide aerogel as an adsorbent were systematically evaluated by adsorption capacity, kinetic, isotherm, and thermodynamic for rhodamine B (RhB) in aqueous solution. Its dependence on other parameters such as solution pH, regeneration performance, and different dyes were also investigated.

## 2. Materials and Methods 

### 2.1. Preparation of Coal-Based Graphene Oxide (CGO) and CGO Aerogel

A high-rank bituminous coal derived from Shanxi Province, China was converted into synthetic graphite via high temperature graphitization treatment at 2800 °C for 2 h. The relevant microstructure features of synthetic graphite can be seen in our previous work, and the degree of graphitization for this synthetic graphite was about 93.02% [46]. Coal-based graphene oxide was prepared through a modified Hummers method using the coal-based synthetic graphite as the starting material. Here is a typical procedure: 1.5 g of coal-based synthetic graphite, 1.5 g NaNO_3_, and 3 g KMnO_4_ were added to 66 mL concentrated sulfuric acid (98 wt %) in sequence under ice bath with continuous stirring for 60 min. Subsequently, the mixture suspension was transferred to 35 °C water bath, maintained under stirring for 30 min, and then slowly diluted by 132 mL deionized water, controlling the temperature at below 85 °C. Afterwards, about 5 mL of H_2_O_2_ (30 wt %) was added into the mixture suspension drop by drop until the appearance turned bright yellow. This yellow mixture was separated by centrifugation (8000 rpm, 6 min), and the remaining solid was subsequently washed with 5 wt % HCl solution and deionized water several times; then, the final resultant solid was ultrasonicated in deionized water for 75 min to obtain a coal-based grapheme oxide (denoted as CGO) suspension.

**Figure 1:**
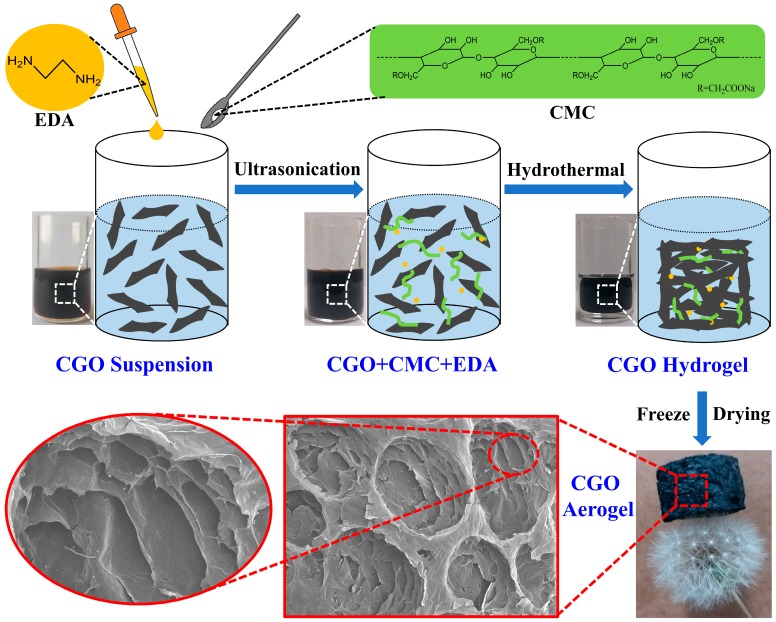
Schematic illustration of the synthesis route for preparing CGO aerogel.

The CGO aerogels were synthesized through a one-step hydrothermal process, and subsequently freeze-dried. Firstly, 10 mL CGO suspension (3 mg·mL^−1^) was homogeneously mixed with a certain amount of carboxymethyl cellulose (CMC) and dispersed by ultrasonication for 30 min; then, 40 μL ethylenediamine (EDA) was added into the mixture solution and ultrasonic treatment was continuously carried out for 30 min. The CGO/CMC/EDA mixture was transferred into a Teflon stainless steel reactor through hydrothermal treatment at 120 °C for 10 h to obtain CGO hydrogel. After that, the CGO aerogel was prepared by freeze-drying the hydrogel at −70 °C for 48 h. The specific schematic illustration of the synthesis route for preparing CGO aerogel is shown in Figure 1. In terms of the different additive amounts of CMC, the prepared CGO aerogel was denoted as CGOA-X, where X represents the mass ratio of CGO to CMC in aerogel samples.

### 2.2. Characterization

The surface morphology of CGO and CGOA were characterized by a Quanta FEG 250 field emission scanning electron microscope (SEM, FEI, Hillsboro, OR, USA). The surface of all samples was coated with gold prior to SEM observation. The X-ray diffraction (XRD) patterns were performed on a D8 advance diffractometer (Bruker, Karlsruhe, Germany) with Cu Kα radiation source (λ = 0.15418 nm), and patterns were recorded at 2θ in the range of 10–30° with a scanning rate of 10°·min^−1^. Raman spectrum of CGOA was recorded from 2000 cm^−1^ to 900 cm^−1^, using a inVia Raman spectroscope (Renishaw, London, UK) with 633 nm laser excitation at room temperature. The surface functional groups of samples were determined by Fourier transform infrared (FT-IR) spectroscopy using a Nicolet Nexus 470 FT-IR Spectrometer ranging (Thermo Fisher Scientific, Waltham, UK) from 4000 cm^−1^ to 400 cm^−1^. X-ray photoelectron spectroscopy (XPS) analyses were also carried out on an Escalab 250Xi (Thermo Fisher Scientific, Waltham, UK) photoelectron spectrometer at ambient temperature.

### 2.3. Adsorption Experiments

The adsorption behavior of CGOA-X was studied by removing RhB under different conditions. A stock solution with a concentration of 1000 mg·L^−1^ was prepared by dissolving RhB in deionized water, and the aqueous solutions of RhB were adjusted to different concentrations by diluting the stock solution. The pH of the solution was controlled by an appropriate concentration of HCI and NaOH solution. The residual concentration of RhB at different time intervals was determined using an UV5200 UV-visible spectrophotometer (Metash, Shanghai, China), and the measuring wavelength is 554 nm. In addition, methyl orange (MO), crystal violet (CV), amido black (AB) and acid red (AR) were also used to evaluate the adsorption performances of CGOA-3 with the same method. All adsorption experiments were performed in a SHA-B temperature-controlled water bath shaker (Zhongda, Changzhou, China) at different temperatures using a glass flask and repeated twice to ensure the accuracy of the results. The adsorption capacity and adsorption efficiency calculated by the following equations:

*q_e_* = (*C*_0_*− C_e_*)*V*/*m*, (1)

*q_t_* = (*C*_0_ − *C_t_*)*V*/*m*, (2)

*R_e_* = (*C*_0_*− C_e_*)/*C*_0_, (3)

where *q_e_* (mg·g^−1^) and *q_t_* (mg·g^−1^) are the adsorption capacity, *C*_0_ (mg·L^−1^) and *C_e_* (mg·L^−1^) are the concentration of dyes before and after adsorption, *C_t_* (mg·L^−1^) is the concentration of dyes at time *t*, *V* (L) is the solution volume, *m* (g) is the mass of the adsorbent used and *R_e_* is the removal efficiency.

## 3. Results and Discussion

### 3.1. Structural Characterization

**Figure 2:**
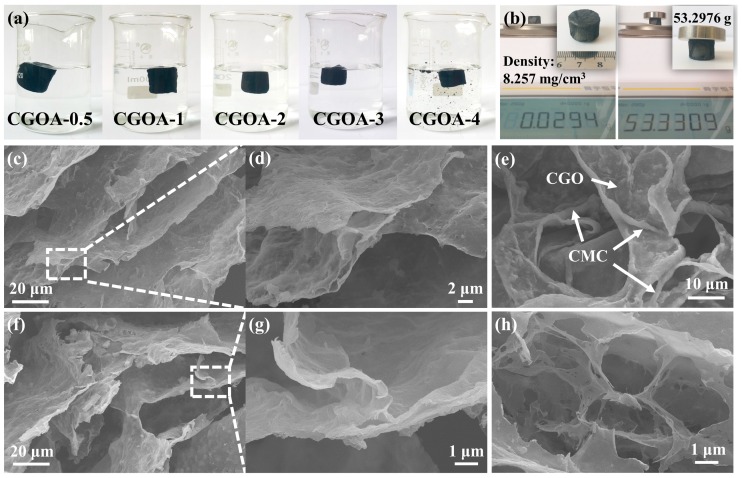
(**a**) Photograph of CGOA after shaking in water for 5 h; (**b**) Density and structural stability of CGOA-3; (**c**–**h**) SEM images of CGOA.

The structural stability and the recovery capacity of the adsorbent in water are necessary conditions for its practical application. Figure 2a is the photograph of CGOA-X (X = 0.5~4) after shaking in water for 5 h. With the decrease of CMC content, the structural stability of CGOAs gradually declines, and the sample is shattered when the mass ratio of CGO to CMC is 4. Therefore, the structure and property of CGOA-3 were systematically investigated. As can be seen in Figure 2b, the apparent density of the obtained CGOA-3 is only 8.257 mg·cm^−3^, which is conducive to recovery in water. One CGOA-3 can support a weight more than 1700 times its own weight with little deformation, which further demonstrates the excellent structural stability of CGOA-3. From the SEM images, it can be seen that CGOA-3 has three-dimensional frameworks with randomly opened macroporous structures (Figure 2c,f), which may provide channels for the migration of dye molecules. At higher magnifications, the layers inside the aerogel are cross-linked to each other, rather than being arranged independently to one another (Figure 2d,g); additionally, the CMC is loaded onto the surface of CGO sheets during the hydrothermal process (Figure 2e), which is the reason why aerogel does not collapse during application. Moreover, it should be pointed out that the macropores of CGOA-3 can also be attributed to the sublimation of ice crystals during freeze-drying (Figure 2h) [47], resulting in an ultra-low density of aerogels.

**Figure 3:**
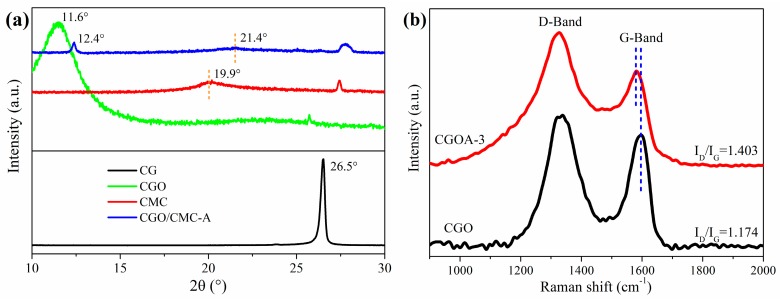
(**a**) XRD patterns of CG, CGO, CMC and CGOA; (**b**) Raman spectra of CGO and CGOA.

The XRD patterns of GO, CMC, and CGOA composites were shown in Figure 3a. The interlayer spacing (d002) of CGO was calculated using Bragg′s law to be about 0.76 nm, corresponding to the diffraction peak at 11.6°. This value is obviously higher than the 0.34 nm for CG (2θ = 26.5°), indicating the successful synthesis of CGO. Compared with CGO, the interlayer spacing (d002) of CGOA was reduced from 0.76 to 0.71 nm, which is attributed to the partial reduction of the CGO sheet during the hydrothermal process by EDA. In addition, CMC shows a broad diffraction peak of its semi-crystalline structure at 19.9°, which suggests that crystallite sizes are very small [42]. After compounding with CGO, the intensity of the characteristic diffraction peaks of CGO and CMC in the composites was significantly weakened, which could be related to the strong interaction between CMC and CGO, resulting in a decrease in their crystallinity. 

Raman spectroscopy is an effective technique for describing the degree of ordering and defects of carbon materials [48]. As can be seen from Figure 3b, both CGO and CGOA shows strong D and G bands, that can be assigned to the structural defects and the vibration of sp^2^-bonded in materials respectively [49]. Accordingly, the intensity ratio of the D-band to G-band (I_D_/I_G_) can be used for the determination of the degree of defects. Compared with traditional graphene oxide [40], a higher I_D_/I_G_ ratio (1.174) of CGO signifies a lower degree of ordering and more defects, which could undoubtedly provide more adsorption sites for dye molecules. After the hydrothermal reaction, the value of I_D_/I_G_ increased from 1.174 to 1.403, probably because the surface of the two-dimensional sheet introduced more heteroatoms from CMC and grafted EDA molecules [47,50]. It should be noted that the G-band is usually shifted when the covalent structure is formed between the carbon material and other components [51]. Therefore, the shift of the G-band in CGOA implies the formation of a new covalent bond in the CGOA.

**Figure 4:**
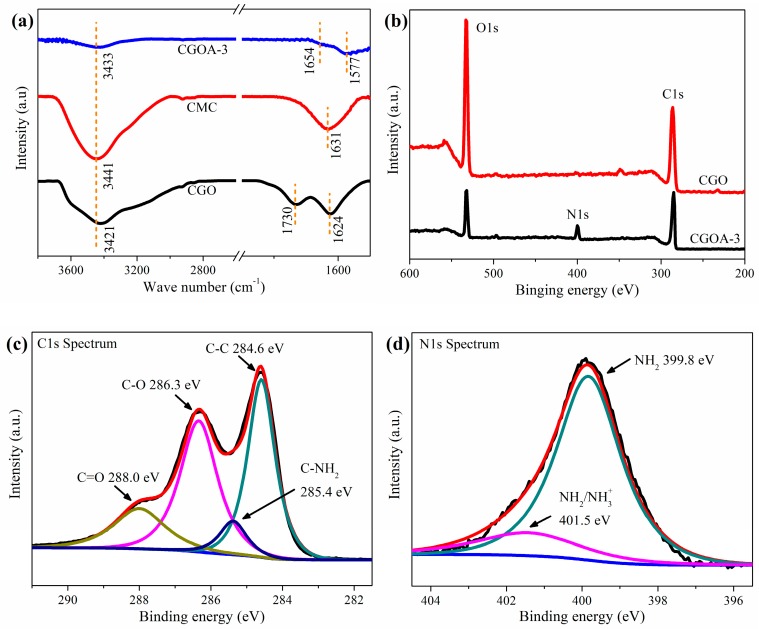
(**a**) FT-IR spectra of CGO, CMC and CGOA; (**b**) XPS spectra of CGO and CGOA; (**c**) Curve fit of C1s spectrum of CGOA; (**d**) Curve fit of N1s spectrum of CGOA.

To further understand the interaction between CMC and CGO, the FT-IR and XPS spectra of several samples are shown in Figure 4. From Figure 4a, the chemical changes in CGOA-3 were investigated with FT-IR spectra before and after the fabrication. In CGO, some characteristic absorption bands appear at 3421, 1730, and 1624 cm^−1^, which represent the stretching vibration of O−H, C=O and C=C, respectively [52]. From the spectra of CMC, peaks at 3441 and 1631 cm^−1^ could be ascribed to the stretching vibration of O−H and symmetrical modes of carboxylate ions [40]. Compared with the CGO and CMC samples, there are several changes for the spectrum of the CGOA-3 sample, which sufficiently verify the strong interaction between CGO and CMC. The stretching vibration of O−H blue shifts to 3433 cm^−1^, which indicates the formation of the composite is facilitated by H-bonding interaction between CGO and CMC. On the other hand, the carbonyl stretch of carboxylate groups of CMC was overlapped with that of carboxylic groups of CGO at about 1577 cm^−1^, which is attributable to the interaction between the hydroxyl groups of CMC and the carboxyl groups of CGO [44,53]. In addition, it is worthwhile mentioning that the new peak at 1654 cm^−1^ is assigned to −NH(R) functional groups [54], suggesting that EDA produced a covalent bond during cross-linking with CGO, in consistent with the Raman analysis.

The surface chemistry of CGO and CGOA were also characterized by XPS. From the comparative analysis in Figure 4b, it can be seen that peaks at ~284.6 eV, ~400 eV, and ~532 eV correspond to C1s, N1s and O1s spectrum, respectively [55]. Among them, the appearance of N1s peaks is ascribed to the cross-linking effect of nitrogen-containing groups (from EDA) with oxygen functional groups of CGO [33]. The C1s spectra can be deconvoluted into four different binding configurations, which include C–C at about 284.6, C−NH_2_ at about 285.4, C−O at about 286.3 and C=O at about 288.0 eV [47]. Similarly, the N1s peak can be deconvoluted into two peaks in the forms of NH_2_ and −NH_2_/NH_3_^+^ [56]. The various oxygen and nitrogen functionalities may endow the aerogel with enhancement of hydrophilicity and plenty of adsorption sites.

### 3.2. Adsorption Performance

**Figure 5:**
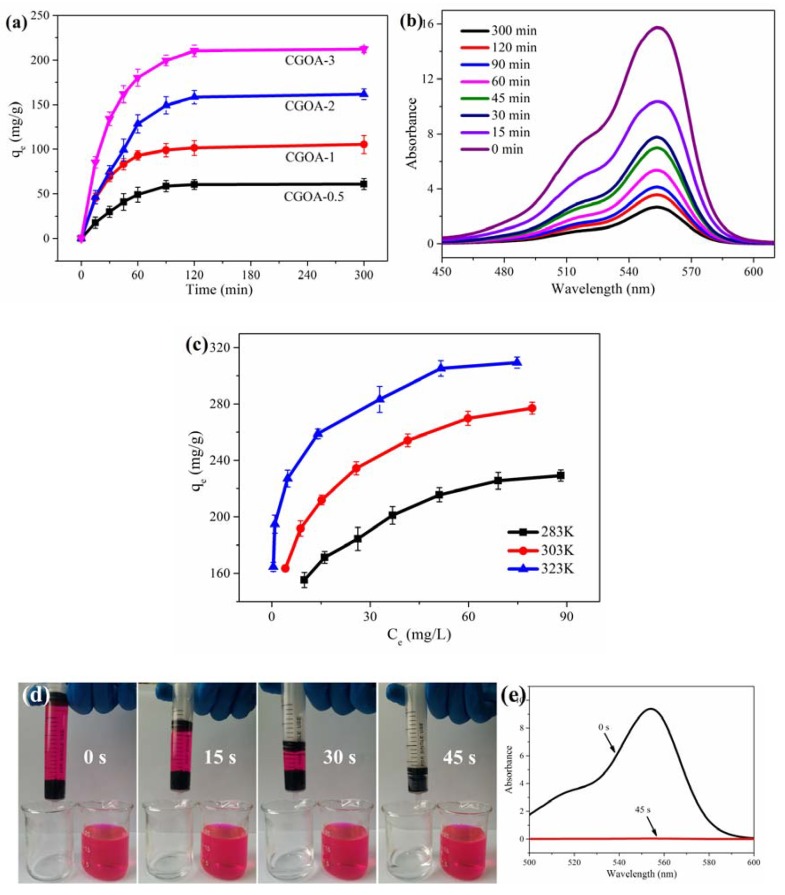
(**a**) Adsorption capacity of CGOA with different CMC content; (**b**) UV-vis spectra of RhB solutions treated with CGOA-3; (**c**) Effect of concentration and temperature; (**d**) Images of the solution before and after filtration; (**e**) UV absorption spectra of the solution before and after filtration.

To compare the adsorption performance of CGOA-X (X = 0.5~3), adsorption experiments were carried out by adding a block of CGOA-X (with the same GO mass, 30 mg) into 50 mL RhB solution with an initial concentration of 100 mg·L^−1^, pH of 10.0, and temperature of 303 K. Figure 5a shows the effect of time on the adsorption capacity of CGOA-X. The adsorption capacity of all samples increased rapidly during the first 60 min, and no obvious change was found after 120 min. Moreover, the adsorption capacity of CGOA-X is gradually increased with decreasing the CMC content, and eventually reaches up to 212.0 mg·g^−1^, which suggests the sample with more CGO possesses much higher adsorption capacity. This phenomenon indicates that the adsorption of RhB primarily occurs on the CGO sheets, and the CMC serves as linker to strengthen the stability of CGOA to maintain the 3D network structure. The UV-vis absorption spectra of RhB in presence of CGOA-3 are presented in Figure 5b. The absorption peak intensity for RhB at 554 nm (the amount of RhB) decreases sharply initially, and then more gently with increasing the absorption time; this is in good agreement with the results presented in Figure 5a. In order to better understand the adsorption process of the dyes, the adsorption kinetic, isotherm, and thermodynamic characteristics of CGOA-3 have been further investigated.

The effects of the initial RhB concentration and temperature of the solution on the adsorption capacity were also investigated at 283 K, 303 K, and 323 K for a given initial RhB concentration ranging from 80 to 140 mg·L^−1^. Figure 5c indicates that the adsorption capacity of CGOA-3 not only increases with increasing concentration of RhB, but also proportionally to temperature. Obviously, the increasing driving force derived from the concentration gradient of the RhB accelerates the diffusion of RhB on CGOA [57], and the adsorbent has more adsorption sites at higher temperatures [58].

Furthermore, the adsorption rate of the adsorbent is also an essential parameter to evaluate its adsorption performance. A 20 mL syringe loaded with a block of CGOA-3 was used to filter the RhB solution of 50 mg·L^−1^ under continuous filtration, as shown in Figure 5d. After 45 s, about 20 mL RhB solution was filtered through the CGOA-3, and the filtrate was collected. The maximum absorption spectrum of RhB disappeared (Figure 5e), indicating that CGOA can remove RhB rapidly and completely.

#### 3.2.1. Adsorption Kinetics

**Figure 6:**
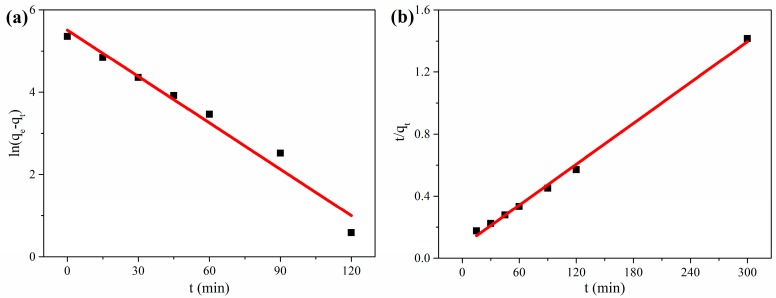
(**a**) Plots of ln(*q_e_ − q_t_*) versus t for the simulation of pseudo-first-order kinetics; (**b**) Plots of *t*/*q_t_* versus t for the simulation of pseudo-second-order kinetics.

In order to predict the adsorption kinetics to RhB, the pseudo first-order model and pseudo second-order model were applied for the experimental data. The pseudo-first-order kinetic model is expressed as [59]:

ln(*q_e_* − *q_t_*) = ln*q_e_* − *k*_1_*t*, (4)

where *q_e_* (mg·g^−1^) and *q_t_* (mg·g^−1^) are the adsorption capacities for RhB at equilibrium and at time *t* (min), respectively, and *k*_1_ (1·min^−1^) is the pseudo-first-order adsorption rate constant. In fact, the values of *q_e_* and *k*_1_ can be obtained from the intercept and slope of the linear plot of *ln(q_e_* − *q_t_)* versus *t* in Figure 6a.

The pseudo-second-order kinetic model is expressed as [60]:

*t*/*q_t_* = 1/*k*_2_*q_e_*^2^ + *t*/*q_e_*, (5)

where *k*_2_ (g·mg^−1^ min^−1^) is the pseudo-second-order adsorption rate constant. Similarly, the plot of *t/q_t_* versus *t* may also yield the values of *q_e_* and *k*_2_ in Figure 6b.

The corresponding kinetic parameters for adsorption of RhB onto the CGOA-3 are shown in Table 1. They show that the pseudo-second-order kinetic curve gives a better fit to the experimental kinetic data compared with the pseudo-first-order kinetic curve; this agreement is certified by higher R^2^ value in Table 1. Moreover, the theoretical value of adsorption capacity determined by the pseudo-second-order kinetic equation is closer to the experimental value, suggesting that the pseudo-secondary adsorption model is suitable for adsorption kinetics of RhB on CGOA.

#### 3.2.2. Adsorption Isotherm

**Figure 7:**
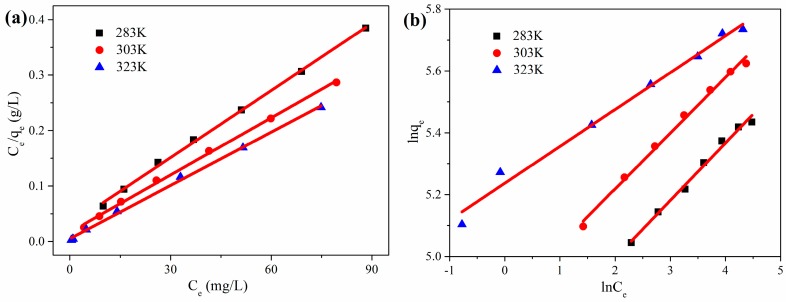
(**a**) Plots of *C_e_*/*q_e_* versus *C_e_* for the simulation of Langmuir model; (**b**) Plots of ln*q_e_* versus ln*C_e_* for the simulation of Freundlich model.

Adsorption isotherm study is one of the most valuable methods to describe the mechanism of the adsorption for RhB, and is essential to predict the adsorption capacity. The Langmuir model assumes that adsorption is confined to a single layer over a homogenous surface with identical adsorption sites, and that there is no chemical reaction between the adsorbent molecules and RhB [61]. The Langmuir model equation can be expressed as:

*C_e_*/*q_e_* = *C_e_*/*q_max_* + (1/*q_max_*)*K_L_*, (6)

where *q_max_* (mg·g^−1^) is the maximum adsorption capacity, *C_e_* (mg·L^−1^) is the equilibrium concentration and *K_L_* (L·mg^−1^) is the Langmuir constant, which is related to the affinity of the binding sites. A straight line with slope 1/*q_max_* was obtained by plotting *C_e_/q_e_* against *C_e_*, as shown in Figure 7a.

For the Freundlich model, it assumes multilayer adsorption over a heterogeneous surface where adsorbed molecules are allowed to interact. The Freundlich model equation can be expressed as:

ln*q_e_* = ln*K_F_* + ln*C_e_/n*, (7)

where *K_F_* and 1/*n* are the Freundlich isotherm constant, and the adsorption intensity respectively, which can be obtained from the intercept and the slope of the linear plot of ln*q_e_* versus ln*C_e_*, as shown in Figure 7b.

Table 2 shows the calculated coefficients obtained from Figure 7 and the Langmuir and Freundlich parameters. By comparing the values of the correlation coefficient *R*^2^, the Langmuir model is more suitable for adsorption data than Freundlich, indicating that RhB adsorption in this system mainly occurred in a monolayer. The adsorption capacity is a pivotal parameter to judge the performance of an adsorbent. The maximum adsorption capacity (323 K) of CGOA-3, as determined by the Langmuir model, is up to 312.50 mg·g^−1^. It can be seen by comparison with other CMC/GO composite adsorbents in Table 3 that CGOA is a competitive adsorbent for dyes removal.

#### 3.2.3. Adsorption Thermodynamic

**Figure 8:**
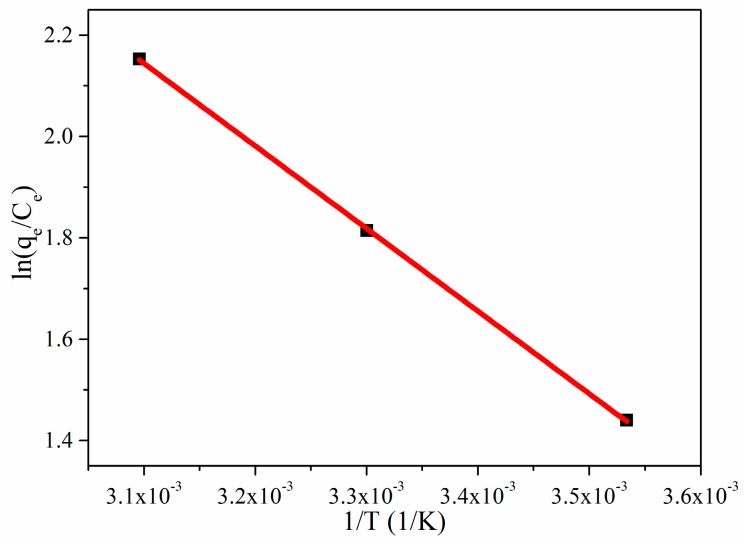
Plots of ln(*K_L_*) versus 1/T for the simulation of thermodynamics.

Figure 8 shows that the adsorption capacity of RhB by CGOA has a certain relationship with temperature. To further evaluate the adsorption mechanism of CGOA, the thermodynamic parameters (Δ*H*, Δ*S* and Δ*G*) are calculated from the following three equations [62]:

ln(*K_L_*) = −∆*H*/*RT* + ∆*S*/*R*, (8)

*K_L_* = *q_e_*/*C_e_* (9)

∆*G* = ∆*H* − *T*∆*S*, (10)

where *K_L_* is the Langmuir equilibrium constant, Δ*H* (kJ·mol^−1^), Δ*S* (J·mol^−1^·K^−1^) and Δ*G* (kJ·mol^−1^) are the enthalpy change, the entropy change and the change of Gibbs free energy, respectively, *R* is the universal gas constant, and *T* (K) is the solution kelvin temperature. Plotting ln(*K_L_*) against *1/T* gives a straight line with slope and intercept equal to −Δ*H/T* and Δ*S/R*, respectively.

The calculated values of thermodynamic parameters are reported in Table 4. As the temperature rises, the value of Δ*G* decreases from −3.3833 to −5.7753 kJ·mol^−1^, indicating that higher temperatures are beneficial to the adsorption process, and that the adsorption is a spontaneous process. Moreover, the positive value of Δ*H* indicated that the adsorption process was exothermic. The positive value of Δ*S* suggested a good affinity of RhB towards CGOA and increased randomness at the solid/solution interface during the adsorption process [63].

#### 3.2.4. Effect of pH, Cycles and Different Dyes

**Figure 9:**
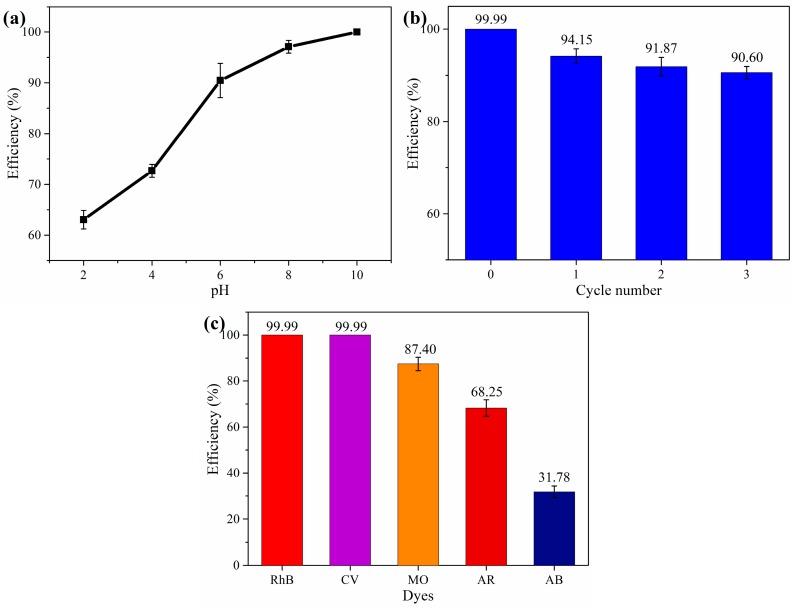
(**a**) Effect of the solution pH; (**b**) Recycling of CGOA; (**c**) Effect of different dyes.

The pH of the solution is one of the important factors affecting the adsorption performance, which can affect the adsorption by changing the protonation of the functional group. The CGOA-3 was added into a 50 mL solution of different pH, the RhB concentration was 50 mg·L^−1^ and the temperature was 303 K. Figure 9a shows the effect of pH values on the adsorption of RhB over the pH range of 2~10. As the pH of the solution increases, the adsorption curve of RB shows an upward trend. RhB is a cationic dye, but high concentrations of H^+^ compete strongly with RhB molecules for adsorption sites in solution, which leads to a lower adsorption capacity. With the constant increase of pH, the competition of H^+^ gradually decreases, and the hydroxyl and carboxyl groups in CGOA will be deprotonated to form −COO^−^ and −O^−^ groups. Therefore, alkaline conditions can enhance the electrostatic attraction of RhB and CGOA, and thus, have a higher adsorption capacity. 

For the desorption study, the above CGOA-3 in RhB solution was collected by filtration, added to a 0.3 M HCl solution, and shaken for 5 h. After that, the CGOA-3 was washed 5 times with 20% ethanol solution to remove residual HCl. At last, the CGOA-3 was regenerated through freeze drying and reused for adsorption with the same conditions. After three cycles of adsorption–desorption, the adsorption efficiency is shown in Figure 9b. It can be clearly seen that CGOA retained 90.60% adsorption efficiency after three reuses. 

MO, CV, AB, and AR have been used in adsorption studies to further explore the adsorption capacity of CGOA for other dyes. The adsorption capacities for different dyes under the same conditions were shown in Figure 9c. Obviously, the adsorption efficiency for cationic dyes RhB (99.99%), CV (99.99%), and MO (87.40%) are much higher than the anionic dyes AR (68.25%) and AB (31.78%). This phenomenon is caused by the strong electrostatic attraction between cationic groups in dyes molecules and the anionic groups of CGOA.

## 4. Conclusions

Graphene oxide aerogel (CGOA) with high mechanical stability was successfully synthesized from bituminous coal and carboxymethyl cellulose by a mild hydrothermal treatment coupled with freeze-drying method. The prepared CGOA possesses a 3D interconnected network structure, beneficial graphene framework defect and abundant oxygen-containing functional groups. The adsorption of RhB molecules on CGOA occurs rapidly through electrostatic interactions. The adsorption kinetics was nicely fitted by the pseudo-second-order kinetic model, which indicates the adsorption rate is limited by the diffusion of RhB molecules onto the CGOA. The adsorption isotherm was best described by the Langmuir model and has a predicted maximum adsorption capacity of 321.50 mg·g^−1^ at 323 K. The thermodynamic analysis suggests that the adsorption of RhB by CGOA is spontaneous and endothermic. CGOA can be effectively regenerated and still retain more than 90% adsorption efficiency after three cycles. CGOA also demonstrated excellent adsorption capacities for cationic organic dyes investigated in this work, and further optimization of the type of materials for dye adsorption is currently underway.

## Figures and Tables

**Table 1:** Parameters of kinetic models.

Kinetic	Parameters	Values
Pseudo-first-order	*q_e_* (mg·g^−1^)	246.6894
	*k*_1_	0.0376
	*R*_1_^2^	0.9682
Pseudo-second-order	*q_e_* (mg·g^−1^)	227.7904
	*k*_2_	0.0002
	*R*_2_^2^	0.9964

**Table 2:** Parameters of Langmuir and Freundlich models.

T(K)	Langmuir			Freundlich		
	*q_max_*	*K_L_*	*R_L_* ^2^	*K_F_*	*n*	*R_F_* ^2^
283	248.14	0.1332	0.9984	101.82	5.3783	0.9889
303	291.55	0.2021	0.9981	128.73	5.5454	0.9938
323	312.50	0.6275	0.9980	188.04	8.3843	0.9851

**Table 3:** Comparison of saturated adsorption capacities of different adsorbents.

Adsorbent	Dyes	*q_max_* (mg·g^−1^)	References
CMC/GO	Methylene blue	59	[44]
	Eosin Y	66	
CMC-AM-GO	Acid Blue-133	185.45	[45]
CGOA	Rhodamine B	312.50	This work

**Table 4:** Parameters of thermodynamics.

*T* (K)	∆*G* (kJ·mol^−1^)	∆*H* (kJ·mol^−1^)	∆*S* (J·mol^−1^·K^−1^)	*R* ^2^
283	−3.3833	13.540	59.780	0.9998
303	−4.5793			
323	−5.7753			
